# Mediating Role of TRPV1 Ion Channels in the Co-exposure to PM2.5 and Formaldehyde of Balb/c Mice Asthma Model

**DOI:** 10.1038/s41598-017-11833-6

**Published:** 2017-09-20

**Authors:** Jing Song, Jun Kang, Bencheng Lin, Jinquan Li, Yuqing Zhu, Junting Du, Xu Yang, Zhuge Xi, Rui Li

**Affiliations:** 1 Section of Environmental Biomedicine, Hubei Key Laboratory of Genetic Regulation and Integrative Biology, School of Life Sciences, Central China Normal University, Wuhan, 430079 Hubei China; 2 Department of Health Toxicology, Tianjin Institute of Health and Environmental Medicine, Tianjin, 300050 China

## Abstract

Asthma is a complex pulmonary inflammatory disease that can be promoted by air pollutants such as PM2.5 and formaldehyde (FA). However, existent experimental evidence principally focuses on the negative influence of a single air pollutant, neglecting the possible synergistic effect in biological responses to mixture of these pollutants, a more common situation in our daily life. In this study, allergic Balb/c mice were exposed to a mixture of PM2.5 and FA, and their toxicological effects and mechanisms were explored. It is demonstrated that the combined exposure to PM2.5 and FA can greatly aggravate allergic asthma in mice. When compared with exposure to PM2.5 or FA alone, the co-exposure showed a certain synergistic effect. Increased levels of ROS, inflammatory factors and total serum immunoglobulin E were concomitant with this deterioration. Furthermore, results suggested that co-exposure exacerbated the activation of TRPV1 signal pathways, with an enhancement in substance P and calcitonin gene-related peptide production, which contributed to inflammation in asthma by neurogenic inflammation. The study also proved that capsazepine treatment could reduce the levels of not only pro-inflammatory neuropeptides, but also oxidative stress. It is concluded that co-exposure to PM2.5 and FA exacerbated allergic asthma through oxidative stress and enhanced TRPV1 activation.

## Introduction

The prevalence of asthma has risen sharply in the world. It is estimated that about 300 million people are affected by asthma, and it is believed that there will be more 100 million people affected by 2025^1^. Asthma, a clinical syndrome comprising intermittent respiratory symptoms, is usually characterized by nonspecific airway hyperresponsiveness and inflammation. It can not only be triggered by virus but also environmental allergens^2^. Allergen-specific CD4^+^ T cells are regarded as pivotal in inflammation, resulting in the infiltration of eosinophils and the activation of mast cells, followed by tissue remodeling, excessive airway mucus secretion, and airway hyperresponsiveness^3^. Though it is well known that immune mechanisms play a critical part in the occurrence and development of asthma, the limited effect of immune treatments suggests the involvement of additional mechanisms and physiological systems in the asthma process^4^.

Recently, evidence has mounted for bi-directional feedback between immunogenic and neurogenic mechanisms in airway inflammation^5,6^. Transient receptor potential vanilloid 1 (TRPV1), a non-selective cation channel, is not only a member of the TRP channel family but also the pivot of almost all neuronal inflammatory signaling pathways^7^. In addition to earlier reports that TRPV1 was widely present on primary sensory neurons, bronchial epithelial cells, smooth muscle cells in the lung and pulmonary dendritic cells, recent findings have indicated that there is the expression of TRPV1 on T cells, and adjusts the activation and the inflammatory capabilities of CD4^+^ cells^8,9^. Numerous studies have shown that the TRPV1 ion channel is associated with the development of asthma^10,11^. A variety of chemical compounds can activate this channel^4^. The activation of TRPV1 caused a lot of calcium to flow inward, leading to the production of pro-inflammatory neuropeptides like tachykinin substance P (SP) and the calcitonin gene related peptides (CGRP)^12^. It has been suggested that SP and CGRP, released by an activated TRPV1 ion channel, contribute to inflammation in asthma by neurogenic inflammation, including the triggering of specific receptors, and the production of additional inflammatory mediators like cytokines, oxygen radicals and histamine. This inflammation induces an increase in vascular permeability, extravasation of plasma and leukocytes, mucus hypersecretion and airway constriction^13,14^.

Oxidative stress and the generation of excessive reactive oxygen species (ROS) plays a critical role in allergic diseases, including allergic asthma^15^. A recent study reports that there is a feedback between TRPV1 activation and ROS production. ROS is a potential endogenous TRPV1 agonist and second messenger of neurokinin signaling in peripheral sensory neurons^16^. Many studies have shown that SP and CGRP, released by an activated TRPV1 receptor, could stimulate NADPH oxidase to induce the overproduction of ROS. This result has been proven for neurogenic vasodilatation, cardiomyocyte contractile dysfunction and ethanol-induced gastric injury^17–19^. However, there is not enough evidence to support this TRPV1 and ROS relationship in allergic asthma, especially in pollutant-promoted asthma.

It is significant to identify the factors that are closely related to increasing the prevalence of asthma. Large-scale epidemiological studies have shown that exposure to air pollutants, such as ambient fine particulate matter (PM2.5) and formaldehyde (FA), increases the risk of asthma and exacerbates established asthma^20–22^. Although air pollution is almost always in the form of a mixture, experimental and epidemiological studies have principally focused on the negative health effects of individual air pollutant, forgetting the health effects of a mixture of contaminants^23^. PM2.5 air pollution is a continuing challenge to public health in China^24^. Wuhan, a typical industrialized city, has elicited a lot of attention domestically, because the levels of ambient airborne PM2.5 are much higher than WHO standards^25^. *In vivo* and *in vitro* experiments have confirmed that PM2.5 can stimulate the differentiation of Th2 cells to increase inflammation in allergic diseases. The mechanism by which this occurs remains controversial^26,27^. FA is one of the main indoor air pollutants. Sources of indoor air FA include furniture, carpets, paints and the use of FA as a disinfectant^28,29^. Animal experiments have found that the stimulating and auxiliary effects of repeated exposure to FA can promote the response to the antigen^30,31^. Past studies have focused on occupational exposure to FA, however, recent evidence suggests that short-term peak exposure to contaminants can also be serious^32^.

There is very little information to be found in the document when PM2.5 and FA are combined, both the explanations on toxicity and related mechanisms. In order to investigate the exacerbating effects of combined PM2.5 and FA exposure, we conducted the experiment on the basis of an allergic asthma mouse model. Capsazepine (Cpz) was used to verify the specific mechanism of TRPV1 in aggravated asthma. We hope that our findings can be helpful in getting an effective new approach to fight allergic asthma exacerbated by environmental contaminants.

## Results

### PM2.5 chemical ingredients

Carbon, inorganic ions, elements, and polycyclic aromatic hydrocarbons (PAHs) are the main ingredients of PM2.5. Table 1 shows the results of main compositions detected in PM2.5 samples. The levels of organic carbon (OC) and elemental carbon (EC) were 56.1 and 6.47 μg/m^3^, respectively. Table 1 also listed the concentration of Cl^−^, SO_4_^2−^, NO_3_^−^ and NH_4_^+^, the results indicated that SO_4_^2−^ (37.3 μg/m^3^) was the major components of inorganic ions in PM2.5 samples. The determined elements included major elements and minor elements. In this experiment, the descending order of the content found in major elements was Si > Ca > Na > Mg > Zn > Al > K > Fe. The concentration of Si (127 μg/m^3^) was the highest, followed by Ca (49.8 μg/m^3^). In this experiment, the descending order of the content found in minor elements was As > Mn > Sb > Pb = Cu > Cr > Ni. As had the highest concentration (2.19 μg/m^3^), followed by Mn (0.13 μg/m^3^) and Sb (0.05 μg/m^3^). Table 1 also listed a total of 14 measured PAHs. The top three were Fluoranthene (0.004 μg/m^3^), Pyrene (0.004 μg/m^3^) and Chrysene (0.002 μg/m^3^).

**Table 1:** Mean concentration (μg/m^3^) of main compositions detected in PM2.5 samples.

	Component	Concentration	Component	Concentration
Carbons	OC(organic carbon)	56.10	EC(elemental carbon)	6.47
Inorganic ions	Cl^−^	1.64	SO_4_^2−^	37.3
	NO_3_^−^	1.15	NH_4_^+^	1.50
	Al	6.20	Fe	4.02
Major elements	Zn	12.10	Ca	49.80
	K	5.76	Na	17.00
	Mg	15.7	Si	127
	Pb	0.02	Cu	0.02
Minor elements	Cr	0.01	Mn	0.13
	Ni	3 × 10^−3^	Sb	0.05
	As	2.19		
	Naphthalene	2 × 10^−4^	Fluorene	5 × 10^−5^
	Phenanthrene	1 × 10^−3^	Anthracene	4 × 10^−5^
	Fluoranthene	4 × 10^−3^	Pyrene	4 × 10^−3^
PAHs	Benz(a)anthracene	7 × 10^−4^	Chrysene	2 × 10^−3^
	Benzo(a)pyrene	1 × 10^−3^	Benzo(k)fluoranthene	5 × 10^−4^
	Benzo(b)fluoranthene	5 × 10^−4^	Dibenz(a,h)anthracene	3 × 10^−4^
	Benzo(g,h,i)perylene	1 × 10^−3^	Indeno(l,2,3-cd)pyrene	3 × 10^−4^

### Effects of combined PM2.5 and FA on serum total immunoglobulin E (T-IgE) levels in the existence of ovalbumin (OVA)

To assess the effects of combined PM2.5 and FA exposure on serum levels, we detected T-IgE in the serum. All OVA-sensitized groups showed increase in T-IgE levels by comparison with the saline group (Fig. 2A). PM2.5 and FA exposure markedly increased the T-IgE concentration by comparison with the OVA-sensitized group (*p* < 0.01). In comparison with the FA+OVA treated group or the PM2.5+OVA treated group, the FA+PM2.5+OVA treated group showed a dramatic growth in T-IgE concentration (*p* < 0.05; *p* < 0.01). As shown in Fig. 2B and C, more mast cells appeared in the OVA-sensitized groups, and sensitization was more potent in conjunction with co-exposure to PM2.5 and FA. The FA+PM2.5+OVA treated group displayed a huge increment in the degranulation of mast cells by comparison with the PM2.5+OVA treated group (*p* < 0.05). By comparing the OVA-sensitized group with the OVA+Cpz treated group, and the FA+PM2.5+OVA treated group with the FA+PM2.5+OVA+Cpz treated group, we can see that the T-IgE and mast cell granule levels decreased significantly when the mice were treated with Cpz (Fig. 2).

**Figure 1:**
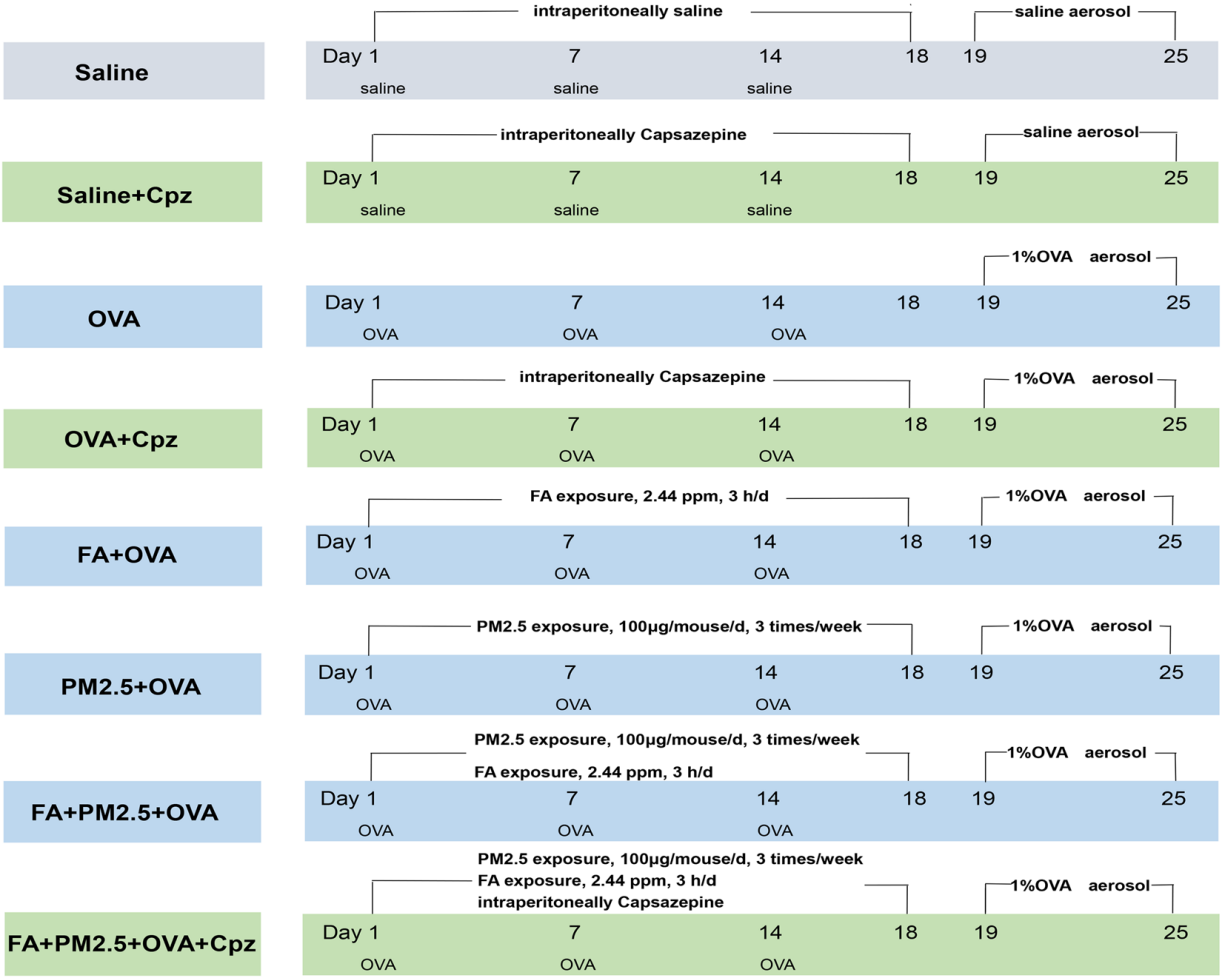
Schematic diagram of the experimental design and animal exposure.

**Figure 2:**
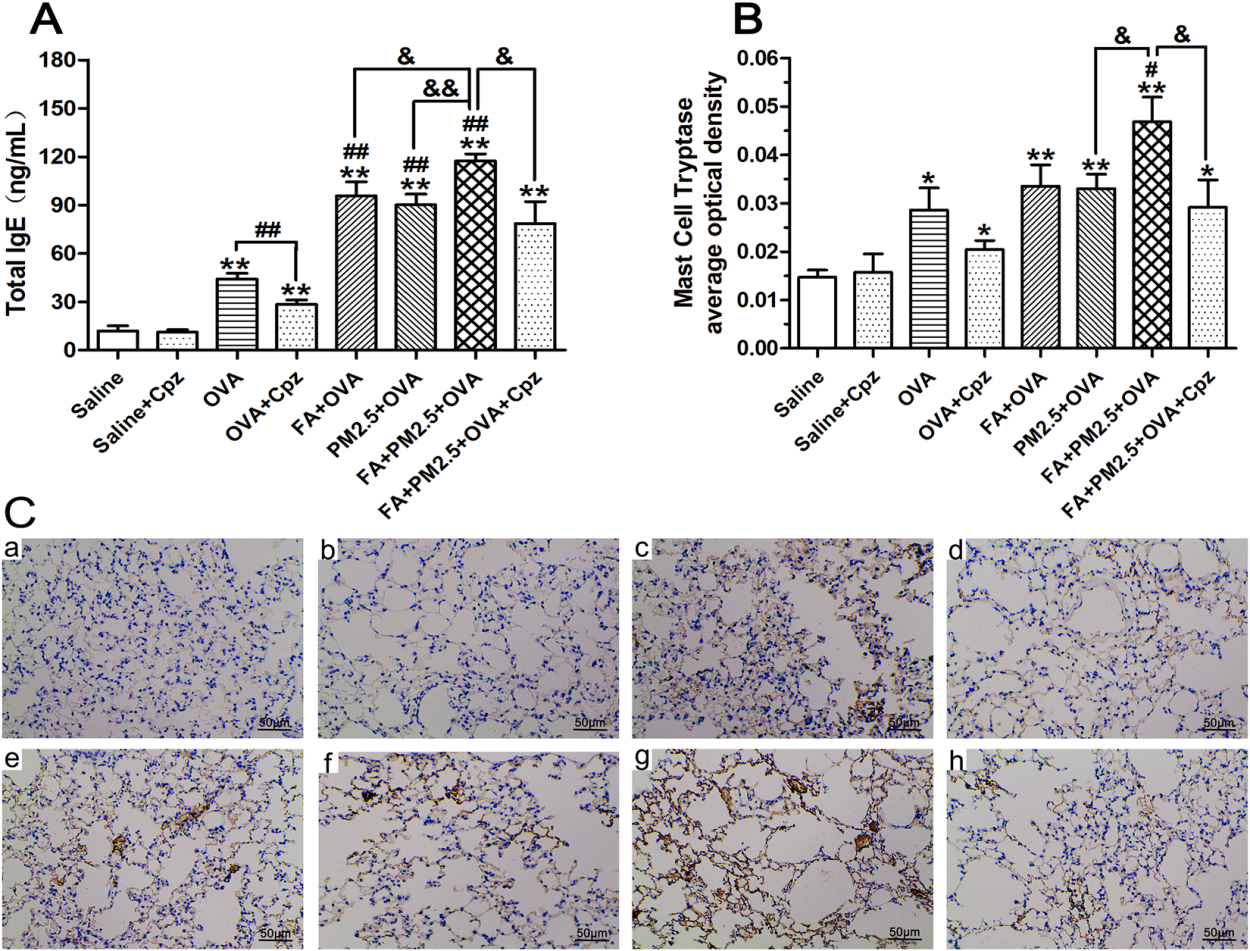
Total serum IgE levels and immunohistochemistry with tryptase. (**A**) Total IgE concentrations (ng/mL). **p < 0.01, compared with the saline control group; ^##^ *p* < 0.01, compared with the OVA-sensitized group; ^&^ *p* < 0.05, ^&&^ *p* < 0.01, compared with the FA+PM2.5+OVA treated group. (**B**) The degranulation scores were calculated from the expression levels of tryptase. *p < 0.05, **p < 0.01, compared with the saline control group; ^#^ *p* < 0.05, compared with the OVA-sensitized group; ^&^ *p* < 0.05, compared with the FA+PM2.5+OVA treated group. (**C**) Immunohistochemistry with tryptase in lung tissue. **B** (a–h) represent different exposure groups (saline, saline+Cpz, OVA, OVA+Cpz, FA+OVA, PM2.5+OVA, FA+PM2.5+OVA, FA+PM2.5+OVA+Cpz), scale bars = 50 μm.

### Effects of combined PM2.5 and FA on the levels of cytokines in Bronchoalveolar lavage fluid (BALF) in the existence of OVA

**Figure 3:**
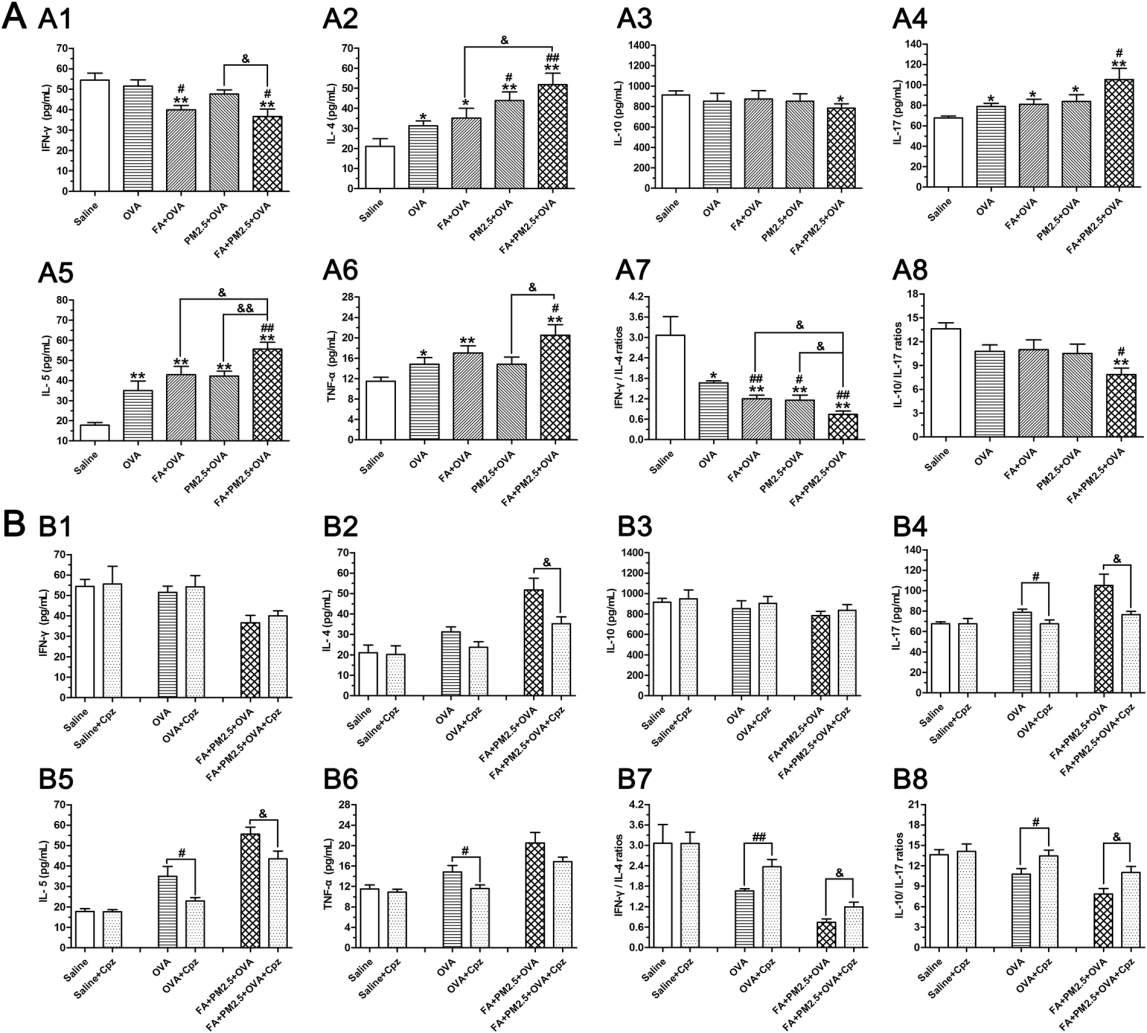
Cytokine levels in BALF. (A1–A8), concentrations of IFN-γ, IL-4, IL-10, IL-17, IL-5, TNF-α, IFN-γ/IL-4 ratio and IL-10/IL-17 ratio in BALF; *p < 0.05, **p < 0.01, compared with the saline control group; ^#^ *p* < 0.05, ^##^ *p* < 0.01, compared with the OVA-sensitized group; ^&^ *p* < 0.05, ^&&^ *p* < 0.01, compared with the FA+PM2.5+OVA treated group. (B1–B8), IFN-γ, IL-4, IL-10, IL-17, IL-5, TNF-α, IFN-γ/IL-4 ratio and IL-10/IL-17 ratio, with protective effects of Cpz in the presence of OVA treatment; ^#^ *p* < 0.05, ^##^ *p* < 0.01, compared with the OVA-sensitized group; ^&^ *p* < 0.05, compared with the FA+PM2.5+OVA treated group.

Interferon-γ (IFN-γ, a Th1 cytokine), interleukin-4 (IL-4, a Th2 cytokine), interleukin-5 (IL-5, a Th2 cytokine), interleukin-10 (IL-10, a Treg cytokine), interleukin-17 (IL-17, a Th17 cytokine) and tumor necrosis factor-α (TNF-α, a proinflammatory cytokine) were measured to confirm the successful establishment of mice asthma model. In addition to the groups applied Cpz, the other groups showed a decrement in IFN-γ and IL-10 concentrations and an increase in IL-4, IL-5, IL-17 and TNF-α concentrations by comparison with the saline control group (Fig. 3A). The ratios of IFN-γ to IL-4 and IL-10 to IL-17 in the FA+PM2.5+OVA treated group were sharply decreased by comparison with the saline control group and the OVA-sensitized group (*p* < 0.01; *p* < 0.05), implying that the Th1/Th2 and Treg/Th17 balances were broken. The FA+OVA treated group displayed a significant drop in IFN-γ concentration compared with the OVA-sensitized group (*p* < 0.05, Fig. 3A1), the PM2.5+OVA treated group showed an obvious growth in IL-4 concentration by comparison with the OVA-sensitized group (*p* < 0.05, Fig. 3A2). The FA+PM2.5+OVA treated group had an enhanced level of IL-5 and TNF-α and a weakened level of IFN-γ in comparison with the PM2.5+OVA treated group (*p* < 0.01; *p* < 0.05). Similarly, combined exposure improved the levels of IL-4 and IL-5 compared with exposure to FA alone (*p* < 0.05). Interestingly, the application of Cpz was seen to reduce the level of TNF-α and the imbalance of the Th1/Th2 and Treg/Th17 immune responses when compared with the OVA-sensitized and FA+PM2.5+OVA treated groups (Fig. 3B).

### Effects of combined PM2.5 and FA on Leukocyte Levels in BALF in the existence of OVA

**Figure 4:**
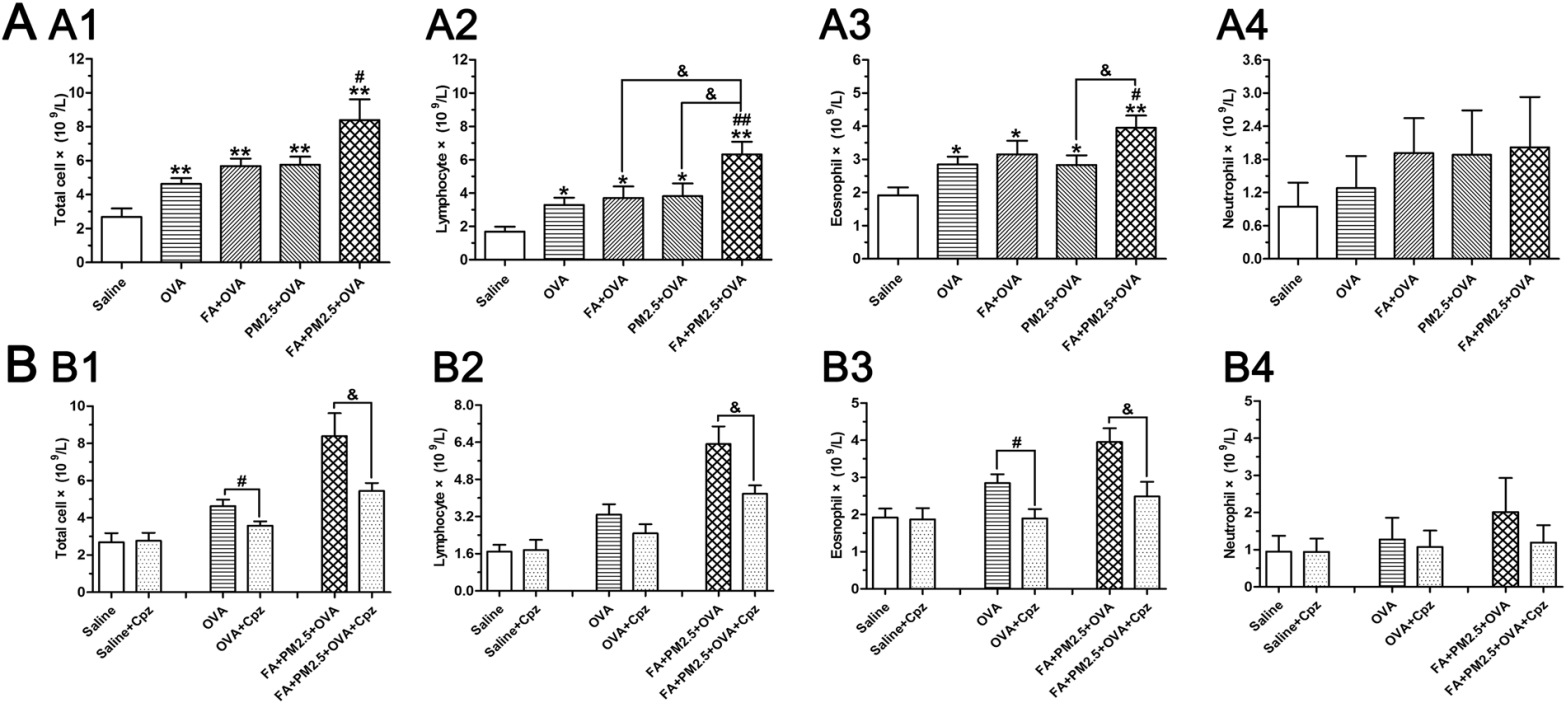
Leukocyte infiltration in the airways in different experimental groups. (A1–A4), effects of combined PM2.5 and FA on inflammatory cell recruitment in the presence of OVA; *p < 0.05, **p < 0.01, compared with the saline control group; ^#^ *p* < 0.05, ^##^ *p* < 0.01, compared with the OVA-sensitized group; ^&^ *p* < 0.05, compared with the FA+PM2.5+OVA treated group. (B1–B4), total cells, lymphocytes, eosinophils and neutrophils, respectively, protective effects of Cpz on inflammatory cell recruitment in the presence of OVA treatment; ^#^ *p* < 0.05, compared with the OVA-sensitized group; ^&^ *p* < 0.05, compared with the FA+PM2.5+OVA treated group.

In this study, the cellular results of BALF showed that treatment with OVA resulted in the clear increase of levels of total cells, lymphocytes, and eosinophils by comparison with those in the saline control groups (Fig. 4A). Combined exposure to PM2.5 and FA showed an obvious increase in total cells, lymphocytes and eosinophils compared with the OVA-sensitized group (*p* < 0.05; *p* < 0.01; *p* < 0.05). Furthermore, combined exposure enhanced the numbers of lymphocytes compared with exposure to PM2.5 or FA alone (*p* < 0.05). In addition to influencing Th2 and Th17 responses, Cpz also played a role in mitigating the aggravating effects of PM2.5 and FA at the cell level. Treatment with Cpz reduced the number of inflammatory cells caused by the presence of PM2.5, FA and OVA (Fig. 4B). The amount of total cells, lymphocytes, and eosinophils in the Cpz treated groups were far less than those in the OVA-sensitized and FA+PM2.5+OVA treated groups, suggesting that Cpz can eliminate airway inflammation to some extent.

### Effects of combined PM2.5 and FA on Airway Remodeling in the existence of OVA

**Figure 5:**
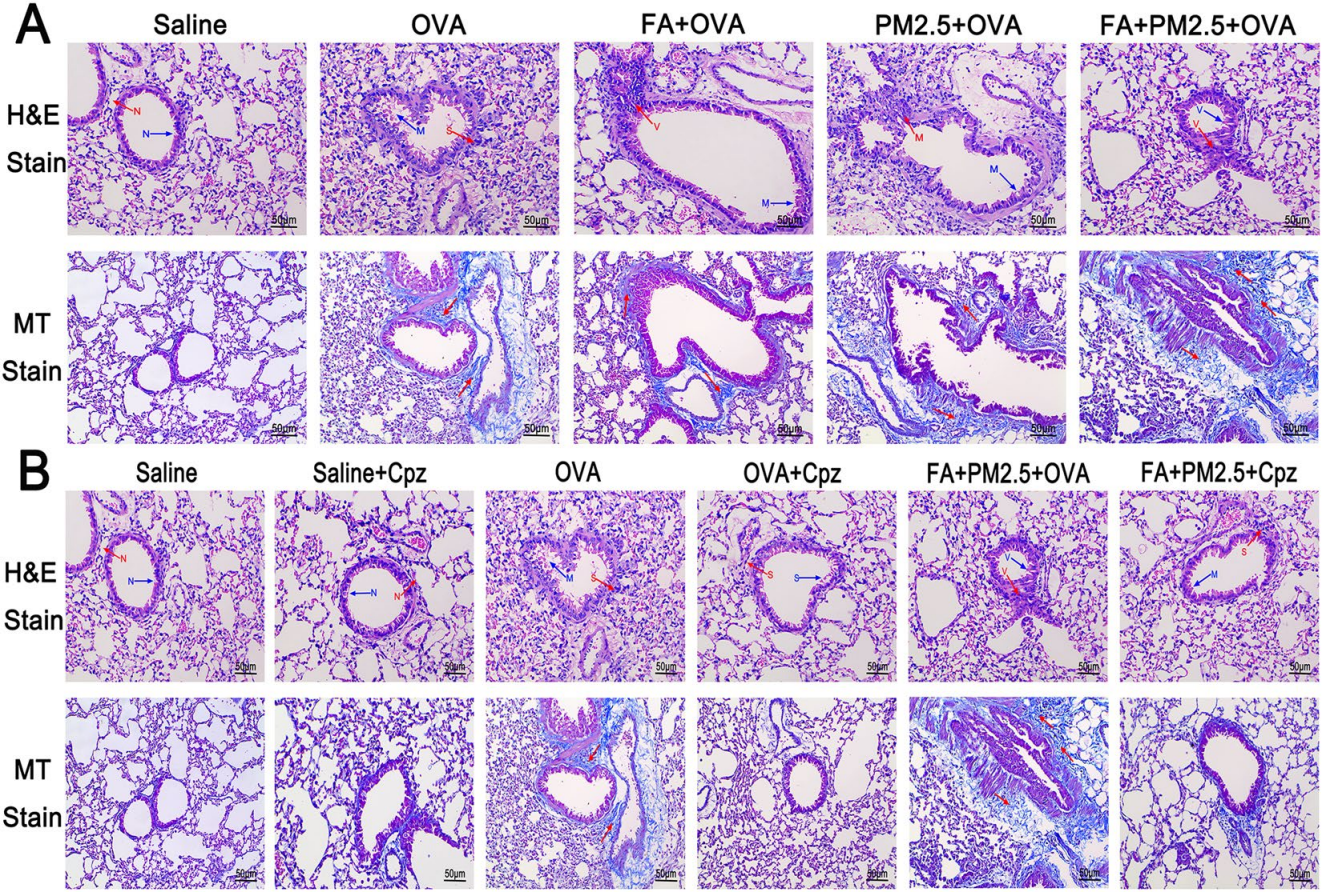
Representative histological images of airway remodeling of lung tissue. (**A**) The effects of combined PM2.5 and FA treatment visualized as histopathological lung changes in the presence of OVA. (**B**) protective effects of Cpz visualized as histopathological changes in lung tissue caused by the presence of OVA treatment. Blue arrow, bronchial remodeling; red arrow, lung tissue cell infiltration; red arrow, subepithelial collagen deposition (blue colored stain); N, S, M, and V indicate normal, slight, medium, and severe changes, respectively.

Increased airway inflammation in mice is closely interrelated with detrimental bronchopathological changes. In practice, the representative bronchopathological characteristics in asthmatic mice is infiltration of inflamed cells in the bronchial region. Hematoxylin and eosin (H&E) staining was used to observe it. Masson’s trichrome (MT) staining was used for observing collagen fiber (Fig. 5A) in OVA-sensitized mice. The results showed that there were slight changes in the OVA-sensitized group in comparison with the saline group and the FA+PM2.5+OVA treated group had the most terrible airway structure changes. The use of Cpz substantially decreased the levels of inflamed cells infiltration and bronchial fibrosis deposition (Fig. 5B).

### Effects of combined PM2.5 and FA on Oxidative Damage in the existence of OVA

**Figure 6:**
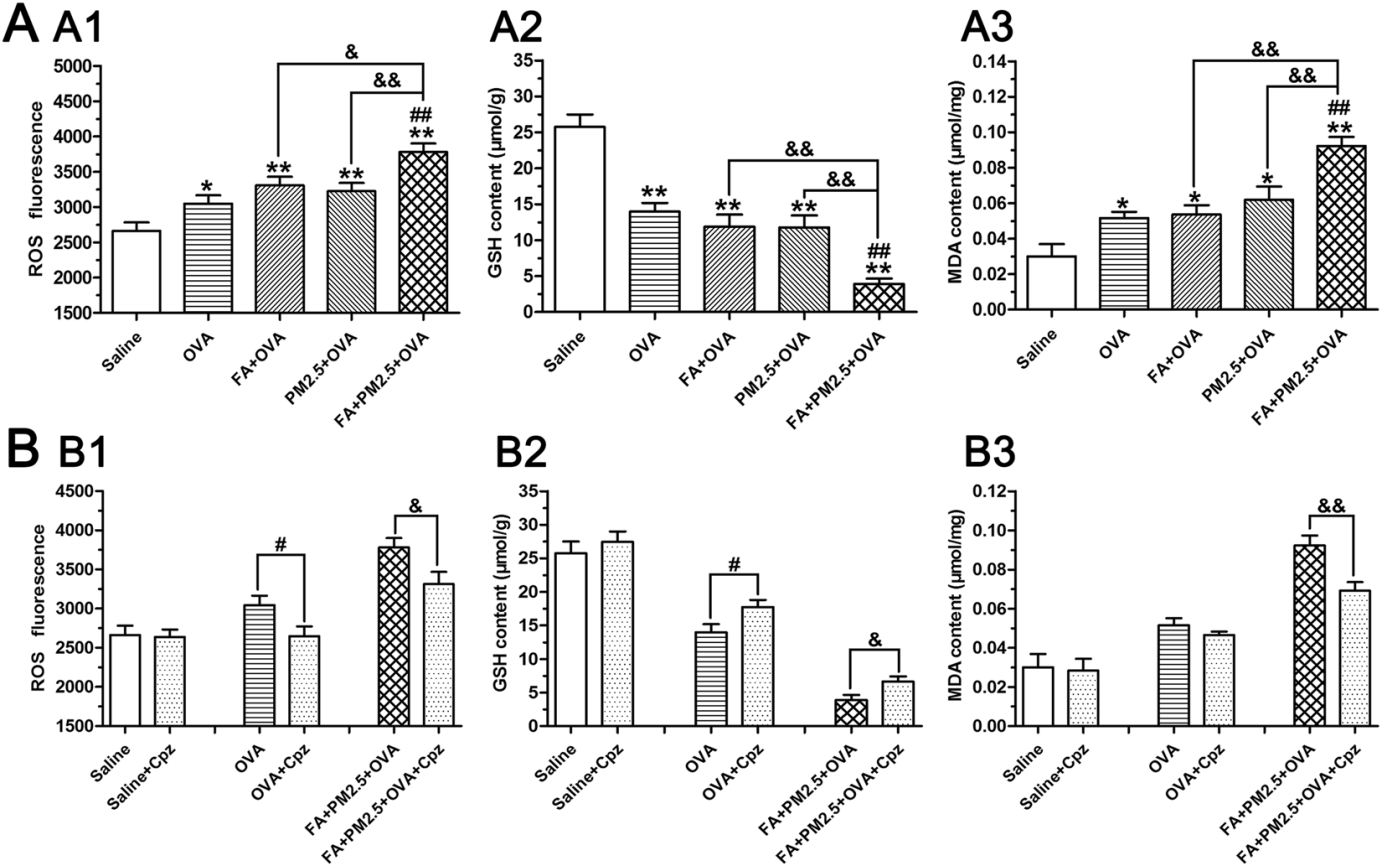
Effects of combined PM2.5 and FA on oxidative stress. ROS, GSH and MDA levels in the lung (A1–A3), and the eliminating effects of Cpz (B1–B3). *p < 0.05, **p < 0.01, compared with the saline control group; ^#^ *p* < 0.05, ^##^ *p* < 0.01, compared with the OVA-sensitized group; ^&^ *p* < 0.05, ^&&^ *p* < 0.01, compared with the FA+PM2.5+OVA treated group.

To assess the level of oxidative damage after FA and PM2.5 exposure, we detected ROS, glutathione (GSH) and malondialdehyde (MDA) in the lung tissues. All OVA-sensitized groups showed an increase in the levels of ROS and MDA and a drop in the level of GSH (Fig. 6A). The levels of ROS, MDA and GSH in the FA+PM2.5+OVA treated group were dramatically changed by comparison with the FA+OVA or the PM2.5+OVA treated groups (*p* < 0.05; *p* < 0.01). This study also showed that intraperitoneal injection of Cpz could inhibit the oxidative stress induced by PM2.5 and FA (Fig. 6B).

### Effects of combined PM2.5 and FA on the Production of Neuropeptide

**Figure 7:**
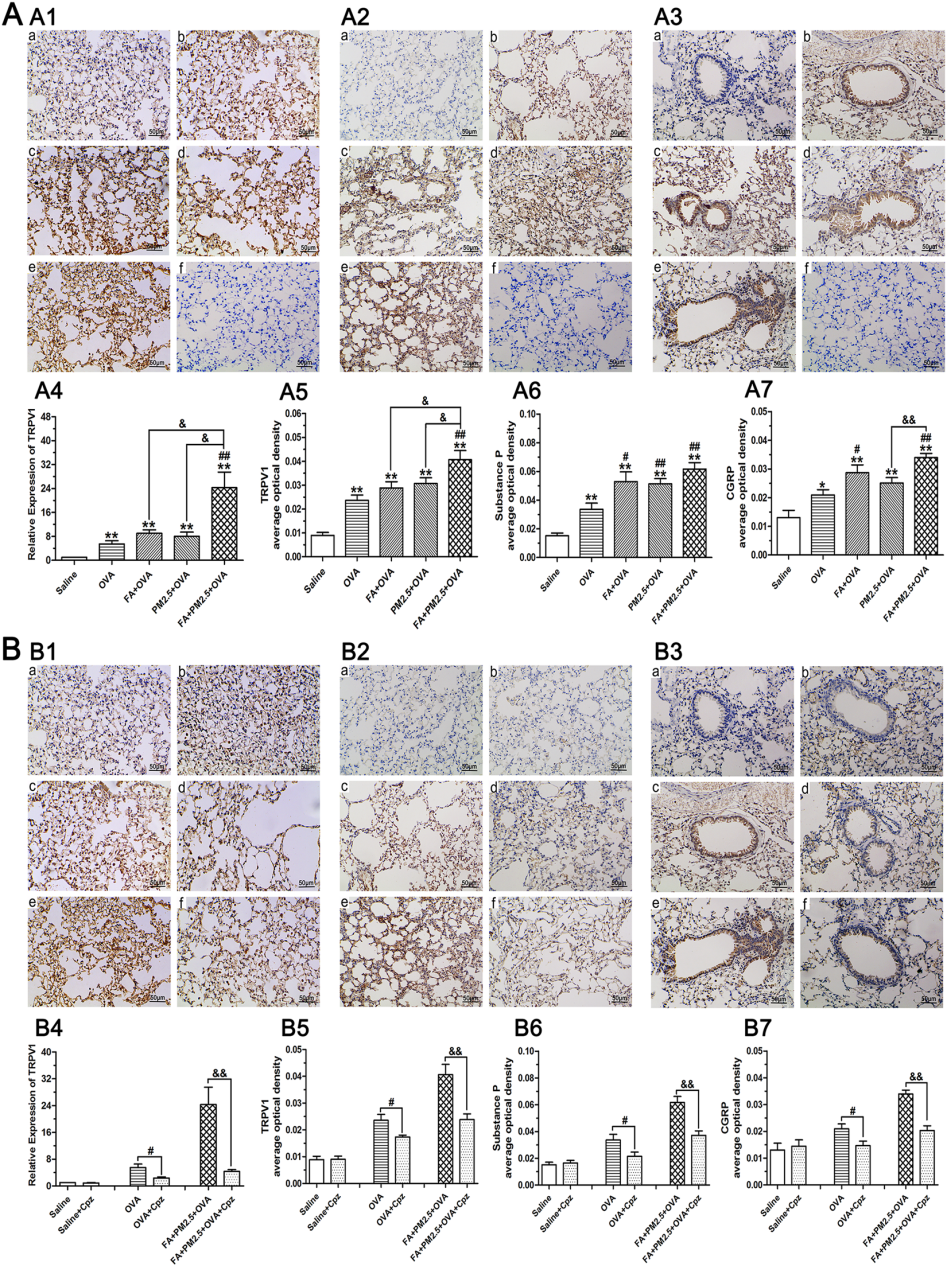
Immunohistochemical and qPCR analyses. (A4) expression of TRPV1 mRNA and (B4) the effect of Cpz treatment on the expression of TRPV1 mRNA. Representative images of the expression of (A1) TRPV1, (A2) Substance P and (A3) CGRP as determined by immunohistochemical staining (brown color stain). (a–f) Represent different exposure groups (saline, OVA, FA+OVA, PM2.5+OVA, FA+PM2.5+OVA, negative control). The eliminating effects of Cpz (B1–B3). (a–f) Represent different exposure groups (saline, saline +Cpz, OVA, OVA+Cpz, FA+PM2.5+OVA, FA+PM2.5+OVA+Cpz). Analyses of (**A5**) TRPV1, (A6) Substance P and (A7) CGRP expression levels and the eliminating effects of Cpz (B5–B7) according to average optical density. *p < 0.05, **p < 0.01, compared with the saline control group; ^#^ *p* < 0.05, ^##^ *p* < 0.01, compared with the OVA-sensitized group; ^&^ *p* < 0.05, ^&&^ *p* < 0.01, compared with the FA+PM2.5+OVA treated group.

The expression of TRPV1 and mRNA level of TRPV1 in lung tissue were detected by immunohistochemical analyses and quantitative real-time PCR (qPCR) (Fig. 7). All OVA-sensitized groups showed an upward trend in TRPV1 expression. Compared with exposure to PM2.5 or FA alone, co-exposure to PM2.5 and FA displayed an obvious increase in TRPV1 expression (*p* < 0.05). Similar to the expression of TRPV1, immunohistochemical analyses of SP and CGRP also showed an increase in the OVA-challenged groups. The expression of SP and CGRP in the FA+OVA treated group showed a significant increase in comparison with the OVA-sensitized group (*p* < 0.05). PM2.5+OVA treated group and FA+PM2.5+OVA treated group displayed a clear enhancement in the expression of SP by comparison with the OVA-sensitized group (*p* < 0.01). The FA+PM2.5+OVA treated group displayed a manifest enhancement in the level of CGRP expression (*p* < 0.01) when compared with the PM2.5+OVA treated group. Levels of TRPV1 were significantly reduced in the OVA+Cpz treated group and the FA+PM2.5+OVA+Cpz treated group as indicated by the results of the immunohistochemical analyses and qPCR analyses (*p* < 0.05; *p* < 0.01). Similarly, the use of Cpz noticeably decreased the contents of SP and CGRP in the OVA+Cpz and FA+PM2.5+OVA+Cpz treated groups (*p* < 0.05; *p* < 0.01).

## Discussion

The target of this study is to reckon the combined toxicity of PM2.5 and FA and the corresponding mechanism on an OVA-sensitized asthma model. The specific policy guidance for health risk assessment of air pollution mixtures is still blank. In this study we confirmed that exposure to PM2.5 combined with FA can increase OVA-caused inflammation and lung tissue injury. By comparison with exposure to PM2.5 or FA alone, co-exposure showed a certain synergistic effect. The results indicated that Cpz, the TRPV1 channel antagonist, reduced these harmful effects, such as the infiltration of white corpuscle, the release of Th2 and Th17 cytokines and T-IgE, the release of pro-inflammatory neuropeptide, the alteration of bronchial structure and oxidative stress. In other words, TRPV1 acts a key part in this combined exposure contributed asthma.

With the increasing severity of air pollution, the potential risk to human health has received increasing attention. What we are concerned about is that in reality, exposure to air pollutants always means exposure to complex mixtures of substances^33^. Therefore, further investigations are needed to determine the toxic effects caused by a mixture of these typical air pollutants.

According to the Wuhan Air Quality Daily published by the Chinese Ministry of Environmental Protection, 9 days were heavily polluted (AQI greater than 200), including 2 days of serious pollution (AQI greater than 300) during our collection period of PM2.5 (10 December 2015 to 11 January 2016). The average concentration of PM2.5 in these days was 206 μg/m^3^ using the formula for AQI and PM2.5 transformation. As we all known, a distinct symptom of asthma is airway inflammation^3^. So the dose we were concerned was the dose of per unit area in the tracheobronchial region. The total air volume of an adult is approximately 500 mL and breathing rate is about 16–20 breaths/min. The deposition rate of PM2.5 in human trachea and bronchus is about 15–16%^34^, the surface area of trachea and bronchi in human and mouse are 0.415 m^3^ and 0.103 m^3^ respectively^35,36^, so the daily deposition dose of per mouse was about 100 μg. Considering there were 9 heavily polluted days in one month, we set a total of 7 times intratracheal instillation in the experimental period. We aimed at the effects of relatively high concentrations of typical environmental pollutants on allergic mice exposed to short-term, so we chose exposure dose for 100 μg/d, 3 times a week, which is similar to the previous studies dose^37,38^.

Studies have shown that high temperature is associated with increasing emission of indoor air pollutants including VOCs and FA. Winter heating or high ambient temperatures can increase indoor FA emissions^39^. Recent studies have shown that FA emissions from the plywood in a confined compartment increased rapidly in the first 3 hours as the temperature rose, and when the temperature rose 5 °C, the equilibrium concentration of FA raised by 1.3 to 2.5 times^40^. The WHO has pointed out that FA concentrations in many pathology laboratories reached 0.18–2.93 ppm, and that plywood workers’ FA exposure concentration averaged 1.2 ppm^29^. Based on these numbers, we set the exposure dose of PM2.5 to be 100 μg/mouse delivered 3 times a week, and the FA exposure dose to be 2.44 ppm for 3 h/day.

Notable features of allergic asthma included the imbalance between Th1 and Th2 immune reactions, the imbalance between Treg and Th17 immune reactions and the production of IgE^2^. The concentrations of IFN-γ, IL-4, IL-5, IL-10 and IL-17 in BALF were therefore determined, to confirm the establishment of the mouse asthma model at the molecular level. In this study, all OVA-sensitized groups displayed a drop in IFN-γ and IL-10 concentrations, but a rise in IL-4, IL-5 and IL-17 concentrations (Fig. 3). Exposure to PM2.5 or FA alone significantly weakened the ratio of IFN-γ to IL-4, and exposure to PM2.5 alone sharply enhanced the production of IL-4 by comparison with the OVA-sensitized group. These results hinted that to some extent, allergic mice exposed to PM2.5 or FA alone, exhibited enhanced lung inflammation. These results are consistent with previous studies^31,41^. The analyses of cytokines in BALF indicated that allergic mice exposed to a combination of PM2.5 and FA produced more serious lung inflammation than exposure to either of the pollutants alone. Furthermore, the ratio of IFN-γ to IL-4 in the combined exposure group was markedly lower than the groups exposed to PM2.5 or FA alone, suggesting that the Th2 response acted a key part in the inflammatory process caused by exposure to both FA and PM2.5. Consistent with the cytokine results, the T-IgE concentrations increased significantly after antigen sensitization. The concentrations of T-IgE were also triggered after PM2.5 and FA exposure in the existence of OVA. The development of antigen challenged airway inflammation can be showed by the levels of serum T-IgE and BALF cytokines, combined PM2.5 and FA exposure aggravated this inflammation.

Airway remodeling is one of the most important factors in asthma, which results in airway narrowing and airflow obstruction^3^. Remodeling of the airways induces structural changes, including fibrosis of the epithelium, hypertrophy of airway smooth muscle and muscular, increased thickness of airway wall and infiltration and activation of eosinophils and mast cells^31^. It should be noted that the recruitment of eosinophils in airway secretions is closely related to the severity of asthma^42^. In this experiment, we used two of the most common staining methods to observe the changes in lung pathology^31^. The degree of lung injury can be shown by H&E staining. After H&E staining, the collagen fibers were red and different thickness. It is difficult to distinguish with other fibers and muscle tissue. That was why we used the MT method to help distinguish and identify collagen fibers^31^. In this study, airway remodeling in the FA and PM2.5 exposure groups were obvious. The FA combined with PM2.5 exposure group showed enhanced histopathological changes in lungs of mice. There was a huge difference when compared with the saline group and the OVA-sensitized group. The cell counting results indicated that the number of eosinophils was dramatically improved in the combined exposure group. Based on these results, we successfully established an animal model of allergic asthma mice and measured the relevant indicators including serum T-IgE, cytokine contents and histopathological changes, in order to judge the role of combined exposure of PM2.5 and FA in asthma.

The results from the immunohistochemical analyses and qPCR suggest that all the OVA-sensitized mice showed an increase in TRPV1, SP and CGRP expression. These results are like the results of other similar experiments^43,44^. Combined exposure to PM2.5 and FA can enhance the expression of TRPV1 compared with exposure to PM2.5 or FA alone, showing a certain synergistic effect. Treatment with Cpz can effectively block the release of these neuropeptides. It may show that allergens enhance the neurogenic immune response by stimulating the production of neuropeptides. Both changed activity of immune cells and degranulation of mast cells are the means by which neuropeptides mediate the immune response. In addition, neuropeptides can influence the production of pro-inflammatory cytokines and oxidative burst by motivate T lymphocyte, macrophages and mast cells^4^. In many tissues, including the lung, release of CGRP can cause vasodilatation and release of SP can not only induce plasma extravasation and bronchoconstriction, but also mucus production^4^. Hence, the production of neuropeptides by activated TRPV1 receptors may act an important role in the growth of asthma by enhancing immune reaction.

ROS plays an active role in the genesis of pulmonary inflammation. GSH, a small molecule composed of three amino acids, is an important antioxidant in the body^45^. MDA is a oxidation product and produced by ROS attacking, which can be used as a sign of oxidative damage. In our study, all the OVA-sensitized mice showed a drop in GSH concentration and an increase in ROS and MDA concentrations. These results are similar to those obtained in previous studies^46^. Co-exposure to PM2.5 and FA led to a significant increase in ROS and MDA levels and a decrease in GSH levels when compared to exposure to a single pollutant (Fig. 6). These results were not seen in animals treated with Cpz. It’s believed that there is a link between ROS and the release of pro-inflammatory cytokines, especially in allergic diseases such as allergic asthma^47^. TNF-α, a typical pro-inflammatory cytokine, is related to oxidative damage. So we tested it in this study. Our data show that the levels of TNF-α detected 24 h after the last challenge, were enhanced in the OVA-sensitized groups by comparison with the saline control group. Co-exposure to PM2.5 and FA resulted in a significant increase compared to the OVA-sensitized group and the PM2.5 alone group. Treatment with Cpz results in a reduction in the level of TNF-α. These results show that co-exposure to PM2.5 and FA can enhance the oxidative stress in the asthmatic mice, and induce the release of the pro-inflammatory cytokine TNF-α, to increase lung damage. In further study it will be important to determine the source of the ROS by using an antagonist of the neuropeptide and antioxidative reagent.

This study suggested that exposure to a combination of PM2.5 and FA can result in increased lung damage in allergic asthma mice by oxidative stress, immunogenic response and neurogenic response. The activation of TRPV1 receptors and the release of SP and CGRP have an important effect on the enhancement of asthma. Cpz, an antagonist of TRPV1, can effectively block this damage.

## Methods

All methods of this experiment were performed in accordance with the approved guidelines and regulations. The testing procedures were approved by the Office of Scientific Research Management of the Central China Normal University on January 24, 2016 (Ratification ID: CCNU-IACUC-2016-003).

### Animals

Male Balb/c mice (5–6 weeks old, 22 ± 2 g) were purchased from the Hubei Province Experimental Animal Center (Wuhan, China). Mice were placed in an independent ventilation cage (IVC) with the standard conditions (12-hlight-dark cycle, 55–75% humidity, and 24–26 °C).

### Main reagents and kits

Capsazepine (CAS 138977-28-3), formaldehyde (4%), and pentobarbital sodium were bought from Sigma-Aldrich (St. Louis, MO, USA). OVA and the ELISA kits (mouse) for measuring IgE, IFN-γ, IL-4, IL-5, IL-10, IL-17 and TNF-α were purchased from eBioscience (San Diego, CA, USA). Rabbit anti-TRPV1-antibody, rabbit anti-SP-antibody and rabbit anti-CGRP-antibody were obtained from Abcam (Cambridge Science Park, UK).GSH was purchased from Nanjing Jiancheng Bioengineering Institute (Nanjing, Jiangsu, China).

### Exposure of gaseous FA

In order to avoid the negative impact of animal fixation on the experiment, we chose the whole-body exposure to FA at 2.44 ppm for 3 h per day in this study. One exposure chamber (Model WH-2, Yuxin S&T Development Co. Ltd., Wuhan, China) was used to achieve the conversion of liquid formalin to gaseous FA. The condition of exposure chamber was stable (2.0 L/min ventilation, 45 ± 5.0% humidity, and 23 ± 0.5 °C), preventing changes in environmental conditions on the experimental results. In the exposure test, the concentration of gaseous formaldehyde was measured every 45 minutes by a FA Analyzer (4160-2, Interscan, Simi Valley, CA, USA).

### PM2.5 sources and characterization

A high traffic total suspended particulates (TSP) sampler (KC-1000, Qingdao) was used to collect ambient air composed of PM <2.5 μm (fine/ultrafine; F/UF) in Wuhan city (113°53′E longitude, 29°58′N latitude), Hubei Province, China. The flow rate of the sampler was 1.2 m^3^/min (±2%) and the sampling time was 8 h per day from 26 November 2015 to 4 February 2016. Carbonaceous aerosols were analyzed using a DRI Model 2001 Thermal/Optical Carbon Analyzer (Atmoslytic Inc., Calabasas, CA, USA). An ion chromatograph (DX-100, Dionex, Sunnyvale, CA) and ICP-AES (61E Trace, Thermo Jarrell-Ash) were applied to test inorganic ions concentrations. The elements in the samples were determined using inductively coupled plasma atomic emission spectrometry (ICP-AES, 61E Trace and ICP-750, Thermo Jarrell-Ash, Franklin, MA). The concentrations of PAHs in the samples were analyzed using the High performance liquid chromatography (HPLC, Hitachi Model 600 HPLC, Hitachi, Japan).

### Preparation of PM2.5 samples

The collected PM2.5 glass filter membranes were cut into small pieces, processed ultrasonically in ultrapure water for 40 min, after which the filter membranes were thrown away. The extracted liquid was vacuum freeze dried, weighted and cryogenically preserved. Before the experiment, freeze-dried particulate matter was mixed with sterile saline to get a particle suspension, which was then subjected to ultrasonic oscillation for 15 min, ensuring that the suspension was uniform and sterile.

### Experimental design and animal exposure

The experiments included 8 groups (n = 6): (1) saline control; (2) Cpz control group (3 mg/kg, intraperitoneal injection); (3) ovalbumin (OVA)-sensitized only; (4) OVA+Cpz; (5) FA+OVA; (6) PM2.5+OVA; (7) FA+PM2.5+OVA; (8) FA+PM2.5+OVA+Cpz. With the exception of the saline control and Cpz control groups, all groups were sensitized with a mixture of OVA+Al(OH)_3_ by intraperitoneal injection on days 1, 7 and 14. The atomized OVA is different from the previous intraperitoneal OVA. The ultrasonic nebulizer was used to finish an aerosol challenge of 1% OVA from days 19 to 25, lasting 30 minutes each time. Cpz was applied as an antioxidant 0.5 h before exposure to FA and PM2.5. Mice were intratracheally instilled with PM2.5 particles of 100 μg/mouse. The detailed protocols are shown in Fig. 1.

### Determination of T-IgE

Pentobarbital sodium was used to treat mice at the end of the exposure experiment, heart blood was obtained through the syringe. Then the samples were centrifuged and the supernatant were stored at −80 °C. The ELISA kit was used to measure the levels of T-IgE according to the manufacturer’s instructions.

### Counts of 4 kinds of Cells in BALF

Followed by the collection of serum was the collection of BALF. The BALF was collected using a syringe with saline. BALF was collected by pressing the chest of mice for 1 minute and the total quantity of each sample was about 1.2 mL. All samples were centrifuged to get supernatant (3000 rpm for 10 min at 4 °C). We used the Blood Cell Analysis system (MTN-21, Matee3nu Technology Corp., Jinan, China) to count the four kinds cells in the cell suspension by suspending the precipitate in BALF with physiological saline.

### Determination of Cytokine Production

After different cell counts, supernatant obtained from the BALF was stored at −80 °C until measurement. The levels of cytokines, including IFN-γ, IL-4, IL-10, IL-17, IL-5 and TNF-α were tested using ELISA kits according to manufacturer’s instructions.

### Determination of Oxidative Stress

The right lungs of the mice were weighed and then homogenized with 10 mL/g ice-cold phosphate-buffer saline (PBS, pH = 7.5) in a glass homogenizer. All samples were centrifuged to get supernatant (10000 rpm for 10 min at 4 °C) and stored at −80 °C. The contents of ROS, GSH and MDA in mice lung tissues were measured just like the previous instructions^48^.

### Pulmonary histopathological assay

The left lungs of the mice were prepared for the histological assay, tissue block size was about 15 × 15 × 5 mm. After washing, dehydration, dipped wax and embedded and some other steps, the slicing machine was set to 5 um thick to get paraffin sections for H&E and MT staining.

### qPCR and analysis

TRIzol method was used to extract total cellular RNA. cDNA synthesis was performed with a high-capacity RNA-to-cDNA Kit (Takara Biotechnology Dalian, China) and reverse transcription was performed as described above. For qPCR, each sample was repeated three times. Light-Cycler 480 software was applied to measure Ct values. The ∆∆Ct method determined relative quantification. The forward and reverse-specific primer sequences of TRPV1 were 5′-AGGCCACTCTTACCACACAG-3′ and 5′-GGCCCAATTTGCAACCAGCTA-3′, the size of the corresponding amplified fragment was 103 bp and the corresponding annealing temperature was 54–57 °C.

### Immunohistochemistry for TRPV1, SP and CGRP

After routinely de-waxing, rehydration and antigen repaired, the 0.3% hydrogen peroxide was used for incubation and appropriate normal serum was used for blocking. Immunohistochemical analysis of TRPV1, SP and CGRP were performed using primary antibodies anti-TRPV1 (1 : 200, Abcam, Cambridge Science Park, UK), anti-SP (1 : 100, Abcam, Cambridge Science Park, UK), anti-CGRP (1 : 50, Abcam, Cambridge Science Park, UK), respectively. Then antibody binding was tested. The reaction product was visualized by diaminobenzidine tetrahydrochloride (DAB) complexes. The negative control was acquired by skipping the primary antibody. After staining, dehydration and hyaline, and mounted sections were mounted in DPX (Sigma-Aldrich). Image-Pro Plus 6.0 software (Media Cybernetics, Bethesda, MD, USA) was used to get the average optical density.

### Statistical analyses

Data are showed as mean ± SEM. GraphPad Prism 5.0 (San Diego, CA, USA) was applied to obtain statistical graphs. A one-way ANOVA, combined with Fisher’s protected t-test was used to determine the significance of differences between the groups. *p* < 0.05 was considered significant and *p* < 0.01 was considered extremely significant.

### Data Availability

All data generated or analysed during this study are included in this published article (and its Supplementary Information files). The datasets generated during and/or analysed during the current study are available from the corresponding author on reasonable request.

## Electronic supplementary material

Supplementary Information
