# Supplementary material for: Mediating Role of TRPV1 Ion Channels in the Co-exposure to PM2.5 and Formaldehyde of Balb/c Mice Asthma Model
Source: Sci Rep. 2017 Sep 20;7:11926. doi: 10.1038/s41598-017-11833-6 (PMC5607312; doi:10.1038/s41598-017-11833-6)
Supplement: Supplementary file 1 — Supplementary Information [file 41598_2017_11833_MOESM1_ESM.pdf]

Mediating Role of TRPV1 Ion Channels in the Co-exposure to PM2.5 and Formaldehyde of Balb/c Mice Asthma Model

Jing Song[1#], Jun Kang[1#], Bencheng Lin[2], Jinquan Li[1], Yuqing Zhu[1], Junting Du[1], Xu Yang[1], Zhuge Xi[2*], Rui Li[1*]

[1]Section of Environmental Biomedicine, Hubei Key Laboratory of Genetic Regulation and Integrative Biology, School of Life Sciences, Central China Normal University, Wuhan 430079, Hubei, China. [2]Department of Health Toxicology, Tianjin Institute of Health and Environmental Medicine, Tianjin 300050, China

*Correspondence to Rui Li (E-mail): ruili@mail.ccnu.edu.cn; Zhuge Xi (E-mail): zhugexi2003@sina.com.

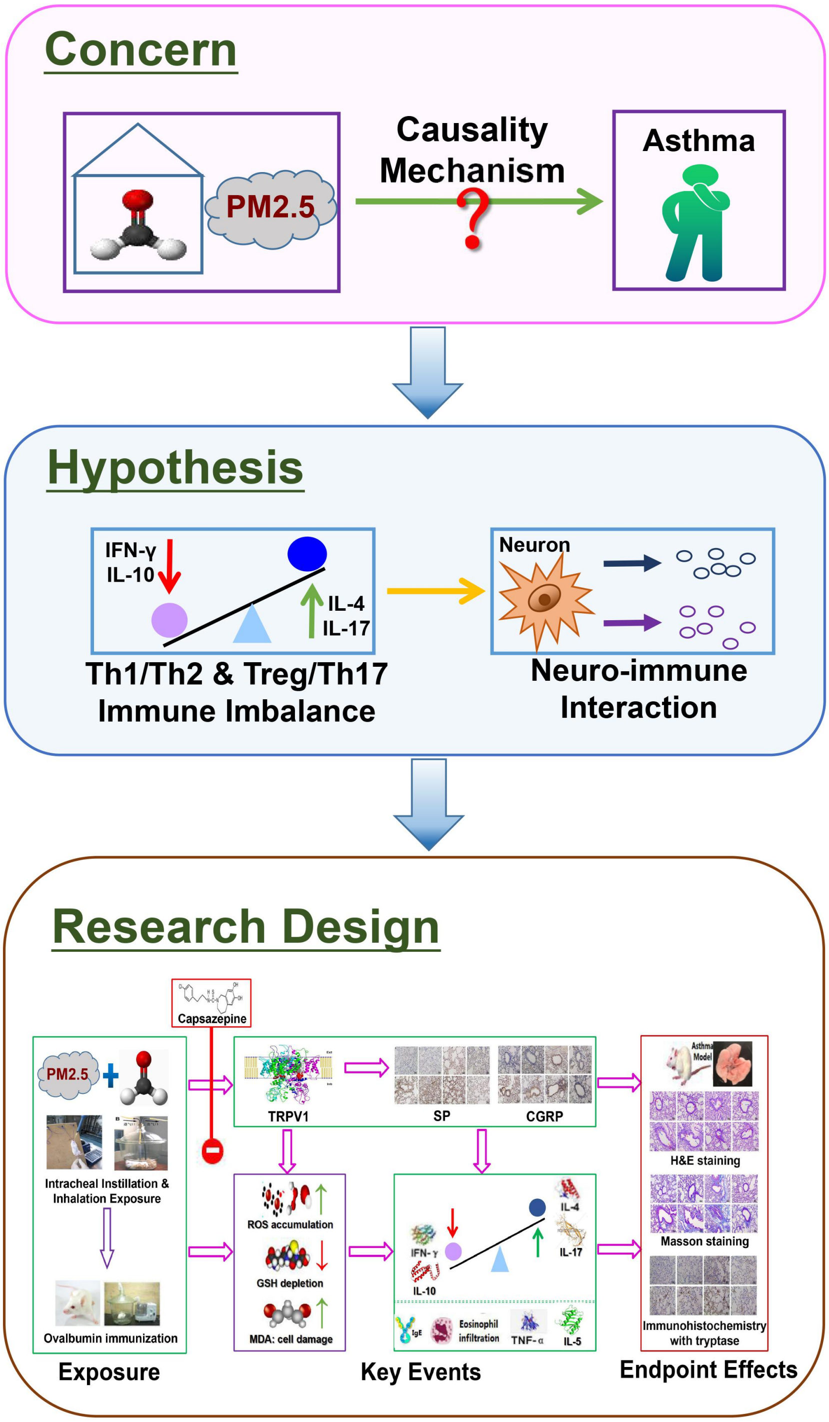

Supplementary Figure S1. Graphical Abstract. (Figure S1 was made by Jing Song, the different photographs of the mice and the photograph of lung were originally taken by Jing Song and the human figure and the drawing of a neuron came from the free software wps http://www.kingsoftstore.com/).
